# Dissecting phenotypic responses of the druggable targetome in cancers

**DOI:** 10.1038/s41598-019-48989-2

**Published:** 2019-08-29

**Authors:** Euna Jeong, Choa Park, Sung Ung Moon, Juyeon Cho, Mee Song, Seungeun Ryoo, Hyejeong Gu, Yejin Lee, Wooyoung Kim, Sukjoon Yoon

**Affiliations:** 1 Research Institute of Women’s Health, Sookmyung Women’s University, Seoul, 04310 Republic of Korea; 2 Department of Biological Sciences, Sookmyung Women’s University, Seoul, 04310 Republic of Korea; 3 College of Pharmacy, Sookmyung Women’s University, Seoul, 04310 Republic of Korea

**Keywords:** High-throughput screening, Targeted therapies

## Abstract

Although a large amount of screening data comprising target genes and/or drugs tested against cancer cell line panels are available, different assay conditions and readouts limit the integrated analysis and batch-to-batch comparison of these data. Here, we systematically produced and analyzed the anticancer effect of the druggable targetome to understand the varied phenotypic outcomes of diverse functional classes of target genes. A library of siRNAs targeting ~4,800 druggable genes was screened against cancer cell lines under 2D and/or 3D assay conditions. The anticancer effect was simultaneously measured by quantifying cell proliferation and/or viability. Hit rates varied significantly depending on assay conditions and/or phenotypic readouts. Functional classes of hit genes were correlated with the microenvironment difference between the 2D monolayer cell proliferation and 3D sphere formation assays. Furthermore, multiplexing of cell proliferation and viability measures enabled us to compare the sensitivity and resistance responses to the gene knockdown. Many target genes that inhibited cell proliferation increased the single-cell-level viability of surviving cells, leading to an increase in self-renewal potential. In this study, combinations of parallel 2D/3D assays and multiplexing of cell proliferation and viability measures provided functional insights into the varied phenotypic outcomes of the cancer targetome.

## Introduction

Together with chemical and drug screening campaigns, functional screening of target genes using RNA interference (RNAi) technologies has been widely carried out to identify and evaluate the therapeutic potential of diverse target genes against cancer cells^1^. Significant amounts of data have accumulated from forward screening using pooled short hairpin RNA (shRNA) or single-guide RNA (sgRNA) libraries against diverse cancer cell lines^2–6^. Although well-based reverse screening using a short interfering RNA (siRNA) library is relatively expensive and thus the amount of accumulated data is limited, it provides the advantage of employing multiplexed phenotypic assays for each of individual gene knockdown^1,7^. We thus attempted to generate well-based screening data for siRNA libraries against four cancer cell lines under two assay conditions with multiplexed readouts.

Most anticancer RNAi (or chemical) screening typically adapts cell viability assays measuring mitochondrial activity or ATP levels in live cells after treatment^8–10^. Specifically, ATP-dependent luminescence (Cell Titer-Glo assay in the Cancer Cell Line Encyclopedia study), fluorescent DNA-binding dyes (SYTO 60 assay in the Cancer Genome Project study) and anionic general biomass stain (SRB assay in the National Cancer Institute study) are used to assess drug inhibition^11–14^. These viability measures are assumed to be correlated with the mass change of cancer cells in the screen, thus representing the anticancer efficacy of treated RNAi. More recently, image-based direct cell counts in two-dimensional (2D) monolayer cultures or sphere counts in three-dimensional (3D) culture conditions have been multiplexed (or employed in parallel) with cell viability assays^15^. In particular, 3D sphere assays have become popular in target screening because they provide an enhanced physiologically relevant microenvironment mimicking *in vivo* tumor formation^16–22^. In our previous study, we identified exclusive anticancer genes that were effective in inhibiting 3D sphere formation but not effective in inhibiting 2D cell proliferation^15^. In the present study, we systematically analyzed the functional diversity of hit genes between 2D and 3D anticancer screens.

Furthermore, we attempted to dissect the phenotypic features in multiplexed readouts (i.e., cell viability vs. cell count measures) on a single-cell basis. The total viability measure per well has been widely used as a surrogate to measure the cell count because the change in the cell number is directly correlated with the total viability measure. However, the viability change in individual cells of the population might be highly varied depending on the anticancer mechanism of the knocked-down gene. For example, the knockdown of cytotoxic genes such as LZTR1 and PLCD3 increased mitochondrial activity and self-renewal potential in the surviving population of cells, but the total cell count decreased^15^. Recent report demonstrated that the varied viability (i.e., mitochondrial mass) in surviving single cells was correlated to the resistant phenotype overcoming the effect of anticancer agents^23^. Thus, the multiplexing of cell count with the change in single cell viability will provide new insights for further dissecting functional classes of anticancer target genes. Here, we report an analytical overview of the functional diversity of target genes displaying diverse outcomes in the multiplexed assays, together with the complete screening data.

## Results

### Inhibitory siRNAs against 2D vs. 3D cancer proliferation

**Figure 1:**
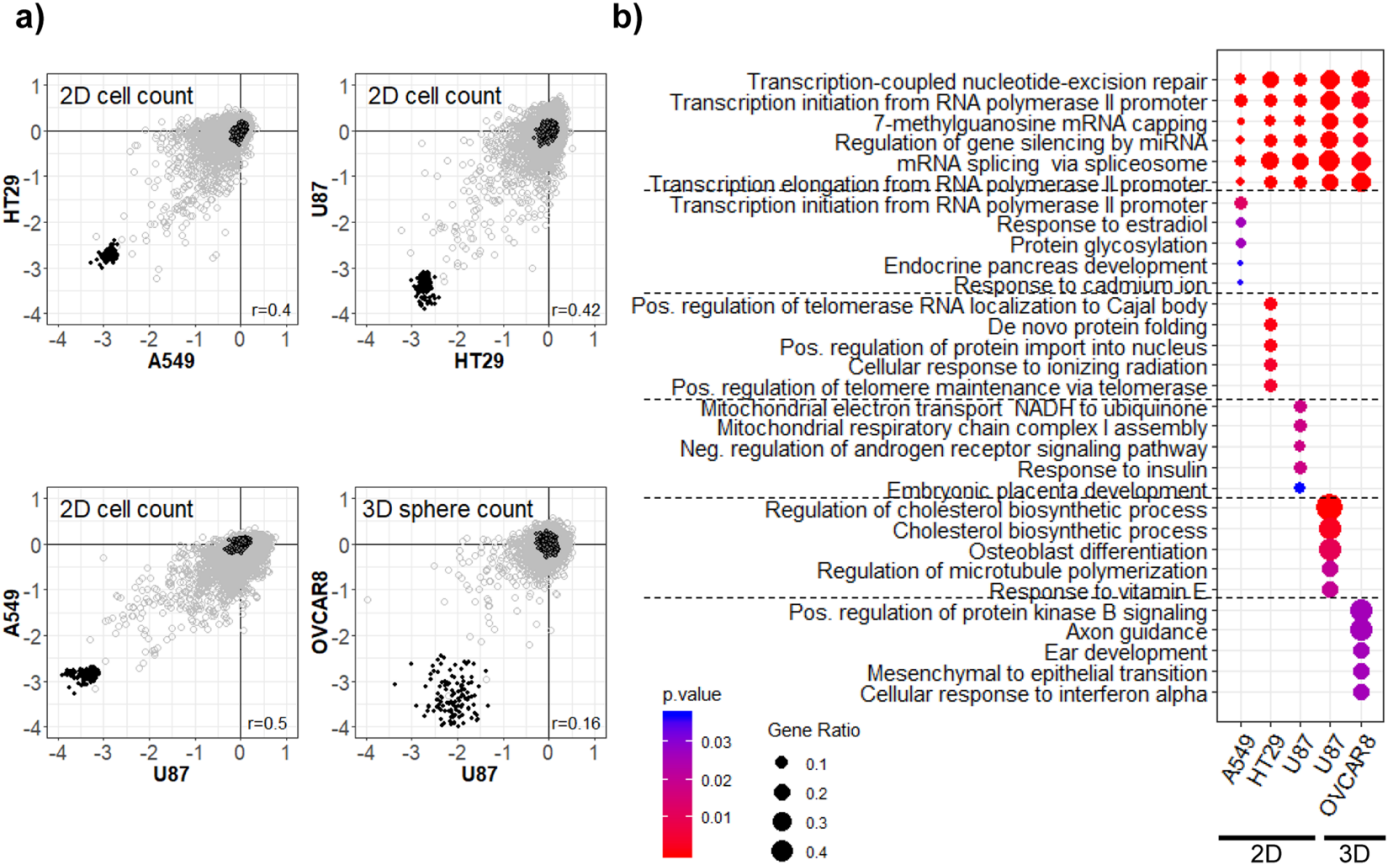
Comparison of screen results from three 2D cell count and two 3D sphere count on four cell lines. (**a**) Screen results were comparatively plotted between samples. Gray circles are the 4,786 siRNAs. The scale of x- and y-axes is log_2_ (fold change) of cell count or sphere count against the control (siNC). Empty and filled dark circles represent siNC and siPLK1, respectively (see Methods section for the detail). The *r* value represents spearman rank correlation of siRNAs between screens. (**b**) Results of gene set analysis were compared among five screen datasets. siRNA hits were analyzed with GO biological process terms. Common and unique gene sets satisfying (two-sided) *p*-value < 0.05 were plotted. Gene ratio is the number of siRNA hits in each gene set divided by the total number of genes in the gene set.

Two measures (i.e., readouts) and two culture conditions were combined for the screening of the siRNA library against 4,789 druggable genes in four different cancer cell lines (Table 1). As shown in Table 1, we observed that overall, 0.5–4% of siRNAs exhibited a significant cancer-inhibition effect (>2-fold change over the control (i.e., siNC) with *p*-value < 0.01) in the screenings. The measure of cell proliferation (i.e., cell counting) under the 2D monolayer culture conditions produced cancer-inhibitory hits in the range of approximately 2.49–4.07%, in comparison to the cell viability measure (approximately 0.56–0.84%) and/or the counting of 3D sphere formation (approximately 0.52–1.15%). We next asked whether the screen results were similar or different between three 2D cell count and two 3D sphere formation assays in Fig. 1. Among the three cell lines (A549, HT29 and U87) from different cancer lineages, the inhibitory hits identified by cell counting showed general consistency (spearman rank correlation: 0.4–0.5), while the consistency of hits inhibiting 3D sphere formation was relatively varied (spearman rank correlation: 0.16) between cell lines (U87 and OVCAR8) (Fig. 1a). The number of common hits between cell lines in each culture condition were 65 (A549 and HT29), 66 (HT29 and U87), and 87 (U87 and A549) for 2D cell count, 15 (A549 and HT29) for 2D viability, and 14 (U87 and OVCAR8) for 3D sphere count. In addition, the common inhibitory hits between 2D cell count and 3D sphere count of U87 was 45 and their spearman rank correlation was 0.35.

**Table 1:** Summary of siRNA hits in diverse anticancer screens.

Cell line	Lineage	Number of inhibitory siRNAs		
		2D monolayer culture		3D culture
		Cell viability	Cell count	Sphere formation
A549	Lung	40 (0.84%)	195 (4.07%)	n/a
HT29	Colon	27 (0.56%)	119 (2.49%)	n/a
U87	Glioblastoma	n/a	141 (2.95%)	55 (1.15%)
OVCAR8	Ovary	n/a	n/a	25 (0.52%)
Sum of unique siRNA hits		302 (6.3%)		

A total of 4,786 siRNAs targeting druggable genes were screened in four cell lines. siRNA hits exhibiting significant anticancer effects (>2-fold change with p < 0.01 in the assay) were counted. The complete list of screening results is available at http://compbio.sookmyung.ac.kr/~siTarget.

n/a = Data not available (experiment was not conducted).

siRNA hits from each of three 2D cell count screening datasets and two 3D sphere formation screening datasets in Table 1 were analyzed with Gene Ontology biological process terms (GO; http://www.geneontology.org) using DAVID (https://david.ncifcrf.gov/). The list of siRNA hits can be found in Supplementary Table 1. Genes belonging to transcriptional activities, such as terms “transcription” and “gene silencing”, were commonly enriched in all five screens (Fig. 1b). We also found cell line-specific enrichment of gene sets such as “transcription”, “protein glycosylation”, “localization”, “protein import”, and “mitochondrial” in 2D screening against A549, HT29 and U87 cell lines. In the 3D screening, lipid metabolic categories such as “cholesterol biosynthetic process” and the protein kinase B signaling category were uniquely enriched in U87 and OVACAR8 cell lines, respectively.

**Figure 2:**
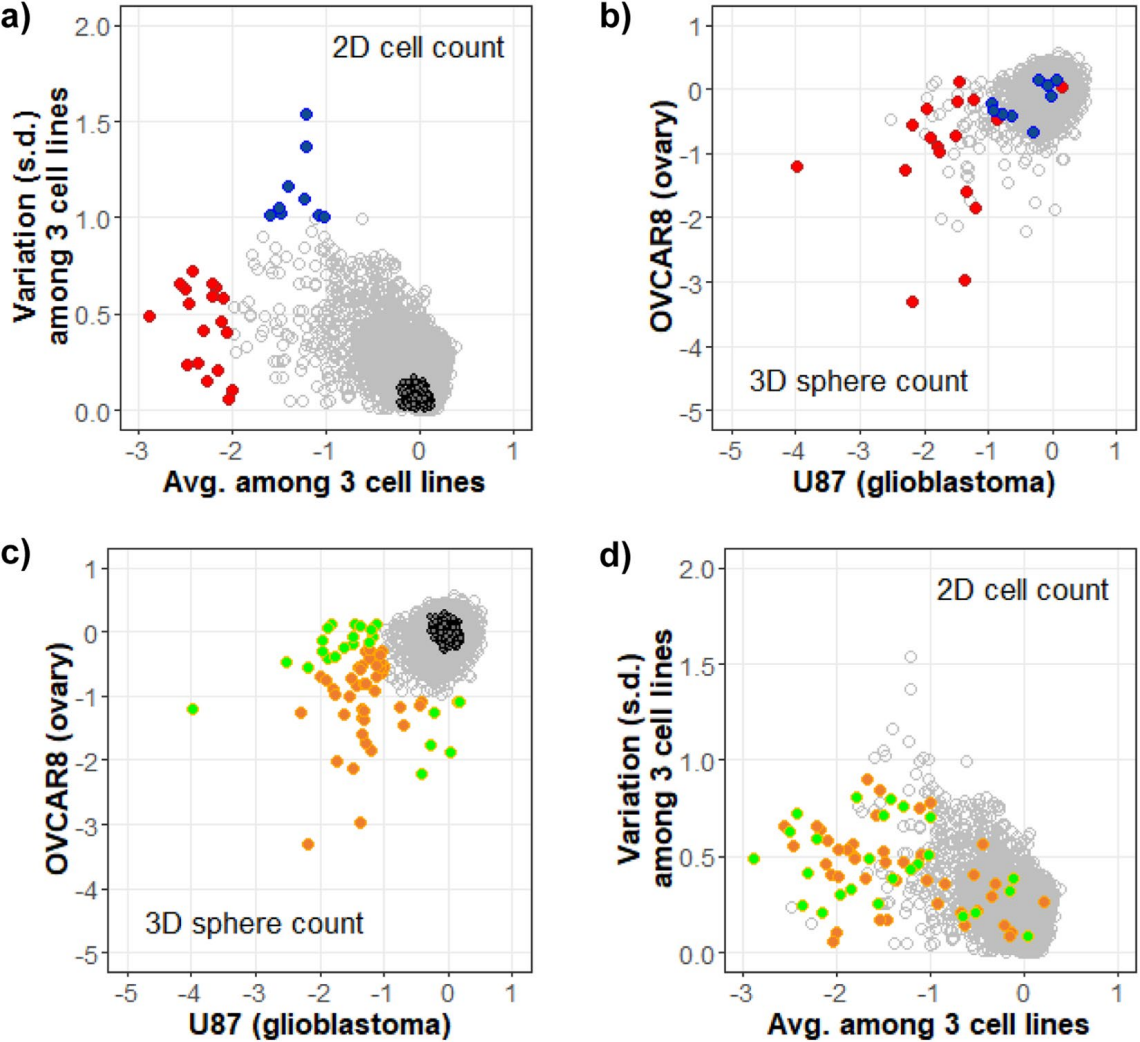
Distribution of inhibitory siRNAs selected by 2D cell count and 3D sphere count. Inhibitory siRNA hits were classified into two groups: sample-independent common hits and sample-selective hits. For the 2D cell count, the average cell count decrease in three cell lines is plotted against the variation of the counts in (**a**,**d**). The results from the 3D sphere counts are plotted between U87 and OVCAR8 samples in (**b**,**c**). The x- and y-axes represent the same as in Fig. 1a in (**b**,**c**). 2D sample-independent (red) and sample-selective (blue) hits are highlighted on the 2D cell count plot in (**a**) and the 3D sphere count plot in (**b**). 3D sample-independent (gold) and sample–selective (green) hits are highlighted on the 3D sphere count plot in (**c**) and the 2D cell count plot in (**d**). Empty dark circles represent siNC in (**a**,**c**). To avoid overplotting, siNC was not drawn in (**b**,**d**).

We further compared the diversity of siRNA hits selected by 2D cell count and 3D sphere count (Fig. 2). The list of selected siRNA hits can be found in Supplementary Table 2. The inhibitory hits from 2D cell counting of three cell lines were classified into two groups in terms of average and standard deviation (s.d.): (1) sample-independent common hits (18 genes) exhibiting a consistent reduction in cell count in all 3 cell lines (average >4-fold change with s.d. <1) and (2) sample-selective varied inhibitory hits (9 genes) (average >2-fold change with s.d. >1) (Fig. 2a). Among 18 sample-independent common hits from the 2D cell count, 15 (83%) and 7 (39%) hits were also effective in inhibiting 3D sphere formation in either U87 or OVCAR8 cells (red in Fig. 2b as log_2_ (fold change) <−1), respectively, while the sample-selective hits from the 2D cell count generally had no efficacy in 3D sphere formation (blue in Fig. 2b). In contrast, a total of 67 siRNA hits inhibiting 3D sphere formation distributed widely in the average vs. s.d. plot of 2D assays (Fig. 2c,d). About 76% of the 67 3D sphere-inhibiting hits were not effective in reducing the 2D cell count in any of the three cancer cell lines as log_2_ (fold change) ≥ −2. This result indicates that genes that inhibit 2D cell proliferation of diverse cell types were generally expected to be effective in reducing 3D sphere formation, while 2D cell proliferation sample-specific inhibitory hits were not effective in inhibiting 3D sphere formation. Only 24% of the 67 siRNA hits inhibiting 3D sphere formation were effective in inhibiting 2D cell proliferation, implying the existence of diversified mechanisms of cancer growth between 2D and 3D culture conditions.

**Figure 3:**
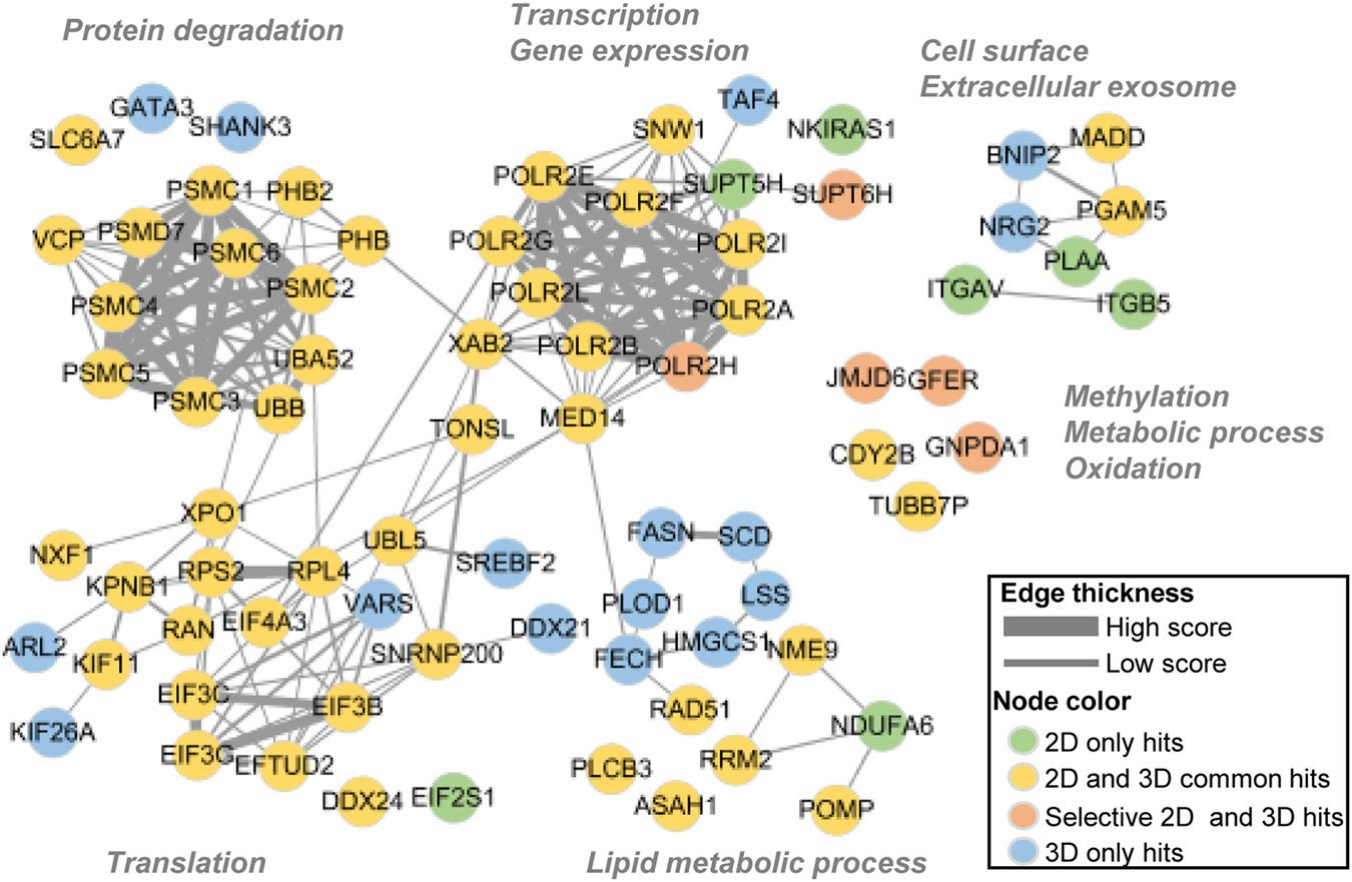
Functional network analysis of selected hit genes from siRNA screens. Gene-gene correlations were plotted using Tanimoto score for 77 siRNA hits. The hits were classified into 2D only hits, 3D only hits, 2D and 3D common hits, and 2D and 3D selective hits. These four classes are colored green, blue, yellow, and orange, respectively. The thickness of the lines between two hit genes represents the extent to which the genes share GO functional terms.

We could thus classify siRNA hits from 2D and 3D screens into four groups as follows: 2D only hits, 3D only hits, 2D and 3D common hits, and selective 2D and 3D hits (Supplementary Table 3 and Fig. 3). A network analysis of these selected genes based on their functional association (see methods section for building network) highlighted the functional separation of the four groups. Out of the seven inhibitory siRNAs exclusively effective under 2D culture conditions, three genes (ITGAV, ITGB5, and PLAA) were found in extracellular exosome and cell-matrix interaction categories (green in Fig. 3). The requirement of cell-plate attachment in the monolayer 2D culture condition might enrich these genes as sensitivity inhibitors for cell proliferation, as they were not effective in inhibiting 3D sphere formation under the low plate attachment conditions. The six siRNA hits exclusively inhibiting 3D sphere formation (FASN, FECH, HMGCS1, LSS, PLOD1, and SCD) were found in cell communication and lipid metabolic process categories (blue in Fig. 3). This observation is consistent with our previous report demonstrating the importance of endogenous regulation of the lipid profile in 3D sphere formation and in the enrichment of cancer stem-like cells^15^. Common hits against both 2D and 3D growth were enriched in essential cellular functions such as transcription, translation and protein degradation (yellow in Fig. 3). Only five siRNA hits exhibited selective inhibitory effects depending on cell type and 2D/3D culture conditions. These selective target genes were found in diverse functional categories, implying the existence of unique anticancer mechanisms for given cancer samples (orange in Fig. 3).

### Varied outcome of target efficacy in cell proliferation vs. viability assays

**Figure 4:**
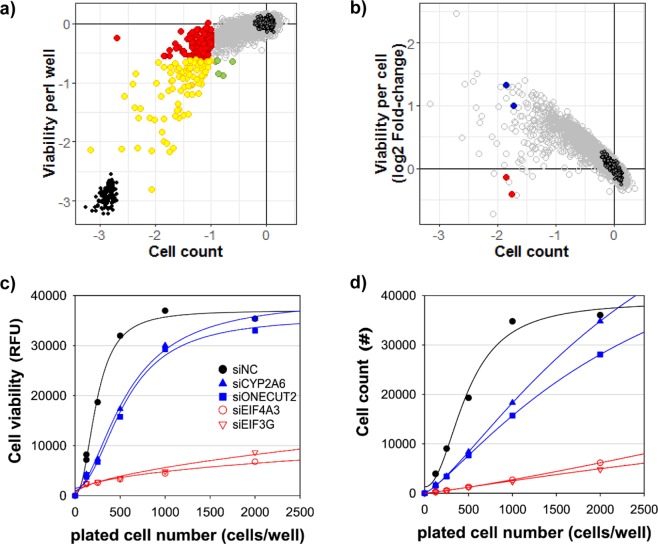
Comparative analysis of siRNA hits between cell count and viability assays. (**a**) 2D cell count vs. 2D cell viability plot of siRNA screens in the A549 cell line. Empty and filled dark circles represent siNC and siPLK1, respectively. Cell count hits, viability hits, and common hits are represented in red, green, and yellow, respectively. The criteria were log2-fold-change <−1 and log2-fold-change <−0.6 with p < 0.01 for cell count hits and viability hits, respectively. (**b**) The total cell count was plotted against the viability per cell using A549 screen data. Two siRNAs (siCYP2A6, siONECUT2) in blue and two siRNAs (siEIF4A3 and siEIF3G) in red were selected for further experiments. (**c**,**d**) Self-renewal ability of A549 cells pretreated with selected siRNAs.

In the screen under 2D monolayer culture conditions, we measured the anticancer efficacy by simultaneously quantifying cell viability (NADPH activity by CellTiter Blue) and cell proliferation (i.e., cell count). Interestingly, the results from the viability measure and cell count showed a low correlation in either the A549 (spearman rank correlation: 0.59; Fig. 4a) or HT29 cell lines (spearman rank correlation: 0.38; Supplementary Fig. 1a). The discrepancy between measures was increased as the inhibitory effects increased (i.e., negative increase of the viability and cell count measure). Among 195 and 21 inhibitory hits obtained from the cell count, 55% and 48% of hits did not show a significant decrease in the viability measure (107 and 10 genes; red in Fig. 4a and Supplementary Fig. 1a), respectively. This observation is consistent with the difference in hit numbers between the two measures in Table 1. Cell counting generated over four times more hits than the viability measure in both A549 and HT29 cell lines. We thus analyzed the change in viability per cell (i.e., average viability of individual cells) in comparison with the change in total cell count (Fig. 4b and Supplementary Fig. 1b). Although siRNA hits inhibiting cell proliferation (i.e., total cell count) exhibited decreased total viability per well (Fig. 4a), their viability per cell was increased (Fig. 4b). Our results indicated that most inhibitory (cytotoxic) siRNA hits in the cell count actually increased the viability of surviving cells in the well. This analysis also explains the observation of fewer hits from the viability measure than from the cell count in Table 1. For example, siRNA treatment against four target genes (CYP2A6, ONECUT2, EIF4A3, and EIF3G) resulted in a similar level of cell count decrease (1.5–2-fold, log-scale) in the A549 cell line screen (Fig. 4b). However, siRNAs against the same four genes showed a large variation (>1-fold, log-scale) in the measure of “viability per cell”. siONECUT2 and siCYP2A6 actually increased the viability of the remaining cells after 72 hours of treatment, while siEIF3G and siEIF4A3 treatment resulted in no significant change (*p*-value > 0.05, in comparison to the siNC treatment) in viability per cell.

**Figure 5:**
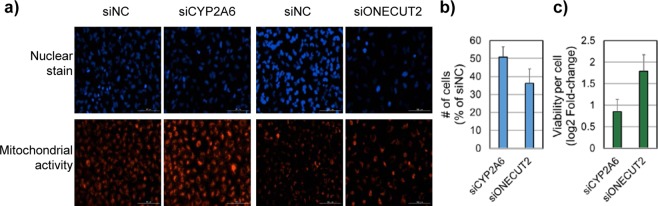
Single-cell-based analysis of mitochondrial activity and cell proliferation. (**a**) Fluorescence image analysis of A549 cells after indicated treatments. Cells were stained with Hoechst 33342 (nuclei, blue) and MitoTracker Deep Red (mitochondria, red). (**b**) Knockdown effect of siCYP2A6 and siONECUT2 in cell proliferation. (**c**) Measurement of single cell viability by MitoTracker DeepRed after treatment with siCYP2A6 and siONECUT2.

To further understand the physiological meaning of the phenotypic disagreement between cell count and viability measure, we carried out a self-renewal assay using these four selected siRNAs in A549 cells. By monitoring the growth of remaining cells in fresh culture media after the treatment of selected siRNAs, we observed that cells treated with siONECUT2 or siCYP2A6 showed greater renewal ability than cells treated with siEIF3G and siEIF4A3 (Fig. 4c,d). This result indicates that the cell count and viability measure may represent different aspects of the anticancer effect. A measure of mitochondrial activity is widely used as a surrogate for the cell viability measure in most cancer screening practices. To further analyze the differential aspects between cell count and viability, we checked the change in single-cell mitochondrial activity during the cell count decrease (Fig. 5) (see Methods section for details). Although the treatment of siCYP2A6 and siONECUT2 decreased the total cell number (Fig. 5b), the mitochondrial activity of individual cells increased significantly (2–4-fold over siNC; *p*-value < 0.001) (Fig. 5c). These opposing outcomes for measures of the anticancer efficacy represent the dual aspect of phenotypic responses (i.e., susceptibility and resistance) of the cancer cell population to the treatment of inhibitory (cytotoxic) siRNAs. This result is consistent with the previous report showing that cells with higher mitochondrial mass were more resistant to the apoptotic stress than cells with lower mitochondrial mass in the clonal cancer cell population^23^.

**Figure 6:**
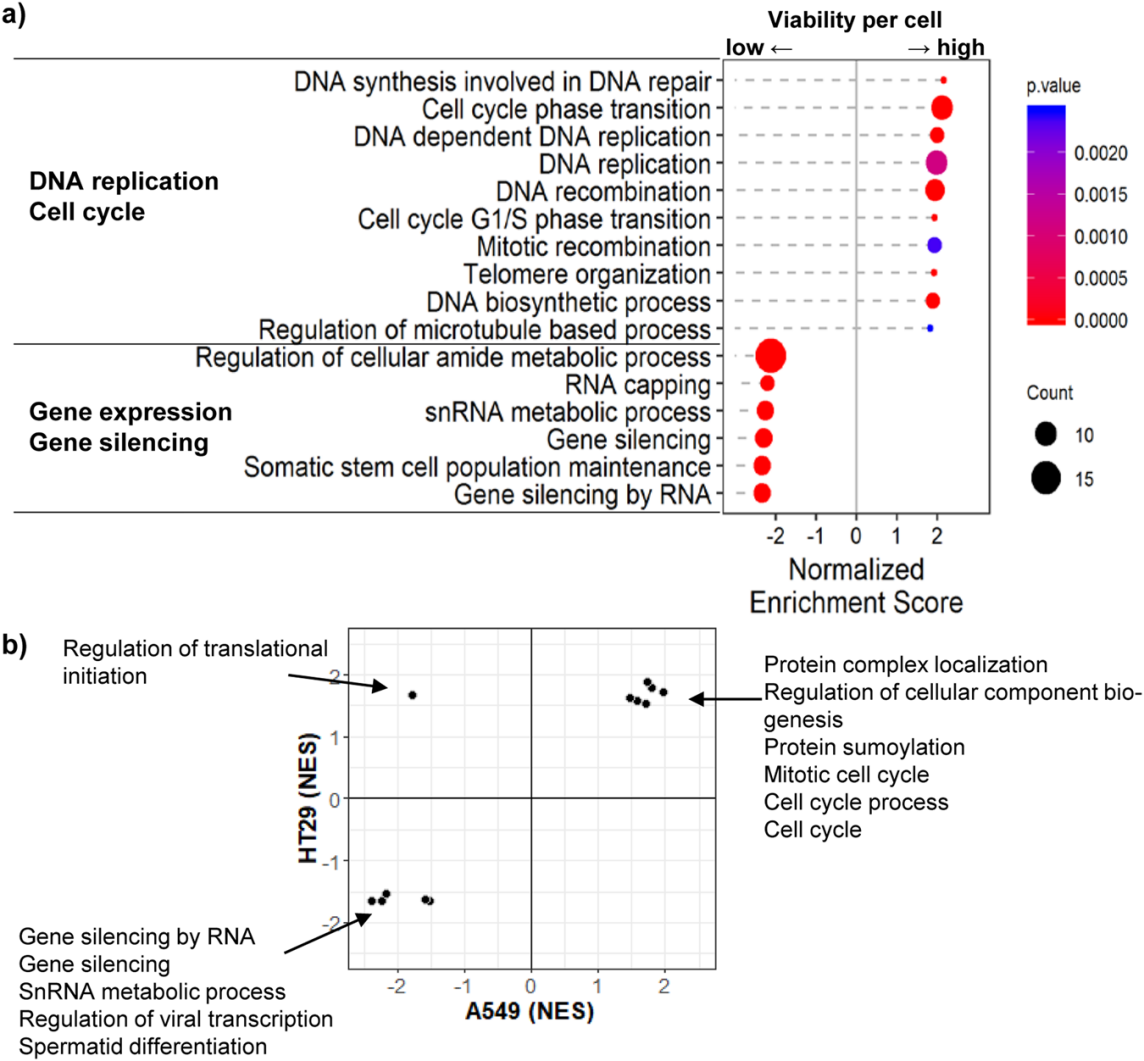
Dissecting functional categories of selected siRNA hits from cell count vs. viability measure. (**a**) We applied Gene set enrichment analysis (GSEA)^25^ to the cell count hits to identify significantly cell count enriched and viability enriched gene sets in the A549 cell line. Genes are ranked by decreasing order of viability per cell measure. Two-sided *p*-value is used for this plot. (**b**) Common gene sets with *p*-value < 0.05 from results of GSEA are plotted between A549 and HT29 samples. The scale of x- and y-axes is normalized enrichment score (NES) from GSEA result.

In the functional gene set analysis of siRNA hits from A549 screening, the cell count assay enriched target genes in DNA replication and cell cycle categories, while the viability measure was less sensitive in prioritizing these hits (Fig. 6a). The functional categories of gene expression and silencing were observed to be relatively more enriched by the viability measure than the cell count. The analysis of hits from HT29 screening showed the enrichment of common function gene sets with A549 (Supplementary Fig. 2 and Fig. 6b). These results show that multiplexing the cell count and viability measures provides a useful strategy for better evaluation of anticancer efficacy in target screening.

## Discussion

Using multiple screening data from the siRNA library of 4,786 druggable target genes, we systematically analyzed the functional classes of anticancer targets based on readouts, assay conditions and sample variation. The screening result and Z’-factor QC data are illustrated in Supplementary Fig. 2. The Z’-factors of 3D assays were lower than that of 2D assays because the number of spheres in 3D were less than the number of cells in 2D assays, resulting in the greater variation among siPLK (control) repeats. The results of functional analysis in Fig. 1b showed that screenings 3D sphere formation enriched differential functional classes of target genes from those in the conventional 2D monolayer screen. Hit variation was significant between 2D and 3D assays, more than between samples in the same assay. Although it has been reported that 3D culture conditions harbor enhanced physiological relevance over 2D conditions^15^, many previously validated target genes were preferentially enriched in the 2D screens rather than the 3D screens in this study. Physiological relevance has critical importance in developing *in vitro* assays for screening anticancer targets. The present study implies that the assay method should be selected based on the functional class (i.e., anticancer mechanism) of target genes.

We also demonstrated that multiplexed assays provided another insight into evaluating the anticancer efficacy of diverse target genes. Convenient cell viability assays are widely used as surrogates for the measurement of cell proliferation in anticancer screening. However, the correlation of a viability measure with cell count was observed to be greatly varied among genes depending on the nature of their anticancer mechanism. The measurement of single cell-based viability (i.e., viability per cell) might represent the resistance response of cancer cells to the anticancer treatment, thus making it too noisy to predict the change of cell mass (i.e., cell proliferation) from the viability measure of total cells in the well. When single-cell-level viability was quantified from the multiplexed (i.e., cell count plus viability) screens, it provided information on both the susceptibility and resistance response of cancer cells to druggable target knockdowns such as CYP2A6 and ONECUT2 genes.

## Methods

### Cells and culture conditions

Four cancer types, including the non-small-cell lung cancer line A549, colon cell line HT29, glioblastoma cell line U87, and ovarian cell line OVCAR8, were used for targetome screening. All cell lines were purchased directly from the National Institutes of Health, National Cancer Institute (NCI, Frederick, MD, USA), except for U87 (American Type Culture Collection, Manassas, VA, USA). All cells were grown in RPMI 1640 (HyClone Laboratories, Logan, UT, USA) supplemented with 10% fetal bovine serum (HyClone Laboratories, Logan, UT, USA) and 1% antibiotics (ThermoFisher, Carlsbad, CA, USA). The cells were cultured at 37 °C under a humidified atmosphere of 5% CO_2_ and 95% air. The culture medium was refreshed every two to three days. We optimized cell seeding number and incubation time based on the previous study^7^.

### High-throughput siRNA screening

The siRNA screen was performed using four pooled On-Target Plus siRNAs to target each of the 4,786 genes in the human drug target library (SmartPools; GE Dharmacon, Lafayette, CO, USA). Screening was performed in triplicate for three and five days in a 384-well plate format for 2D monolayer and 3D culture assays, respectively. Each plate was supplemented with negative control siRNAs (siNC; GE Dharmacon) and positive control siRNAs (siPLK1; GE Dharmacon) to confirm distinguishing sequence-specific silencing from non-specific effects and efficiency of knockdown, respectively. Reverse transfections were performed using a MultiFlo-microplate dispenser (BioTek Instruments, Winooski, VT, USA) with siRNAs (final concentration 10 nM) and Lipofectamine RNAiMax (0.05 μl per well; ThermoFisher Scientific, CA, USA) diluted in Opti-MEM I (Thermo Fisher Scientific) in black 384-well plates (Corning, Corning, NY, USA). After incubation, the cells were stained with CellTiter-Blue for the Cell Viability Assay (Promega, Madison, WI, USA) and Hoechst 33342 for cell counting. The control experiment using siPLK1 against PLK1 (Polo-Like Kinase 1), a gene important for the cell cycle showed that the assay was appropriate for identifying essential genes^24^.

### 2D cell viability and cell counting assay

After high-throughput siRNA screening, the cells were assayed using the CellTiter-Blue Cell Viability Assay kit (Promega) according to the manufacturer’s protocol for the cell viability assay. Hoechst 33342 staining was used to calculate the cell count after fixing cells with 4% paraformaldehyde. The plates were imaged on a Cytation™ 3 (BioTek Instruments) in 2 × 2 montage mode with a 4x objective to capture the entire well.

### 3D sphere formation assay

3D spheres were cultured in serum-free conditioned medium containing 20 ng/ml epidermal growth factor (EGF), 20 ng/ml fibroblast growth factor (FGF) and B27 supplement in DMEM/F12 (all from Invitrogen, Grand Island, NY, USA). After three days, the cells were stained with Hoechst 33342. The plates were imaged using the same method as the 2D cell viability and cell counting assays. The number of cell spheres with diameters greater than 80 μm and 100 μm was counted using Gen5 data analysis software (BioTek Instruments) for OVCAR8 and U87, respectively.

### Screening data analysis

The inhibitory effect of each siRNA was calculated as the log_2_-transformed of readouts: cell count, sphere count, and cell viability compared to the negative controls per plate. After normalization, a negative value means that a readout is more decreased than negative controls. All data units are log_2_-transfomed, unless stated otherwise. Statistical significance was calculated by the one-sided two-sample Student’s *t*-test because siRNA hits were tested for statistical significance in the decrease direction. Viability per cell was calculated by total viability divided by total cell count per well (i.e. per gene). Single-cell-level viability means viability per cell in this study.

### Hit selection

Inhibitory siRNA hits were selected by 2D cell count and 3D sphere count assays. The average inhibitory effect and corresponding variation of three cell line screens (A549, HT29, and U87) were used for selection of 2D count hits. For 3D screens, because there are no specific patterns in terms of average and variation, the screen results of two cell lines were used for analysis. Totally 77 siRNA hits are selected from 2D cell count and 3D sphere count screens. Then, we classified 77 siRNA hits into four groups: 2D only hits, 3D only hits, 2D and 3D common hits, and selective 2D and 3D hits (Supplementary Table 3). From the 2D screens, we selected seven genes that had less than −1 mean cell count and more than 1 standard deviation as 2D only hits. We defined 3D only hits as siRNAs specifically inhibitive in two cell lines (U87 and OVCAR8) and observed 16 genes with a difference between 3D sphere counts greater than 1, and a *p*-value less than 0.01. 2D and 3D common hits were genes with both strong siRNA inhibition in 2D and 3D screens but not sample-specific inhibition. We identified 49 genes as 2D and 3D common hits. Among the three group hits, five functionally unrelated genes were classified as selective 2D and 3D hits.

### Functional analysis and network visualization of hit genes

For the 77 siRNA hits from the 2D and 3D screens, we calculated the functional similarity between every pair of genes based on the Gene Ontology annotations of genes, called the Tanimoto score. We first defined two data sets each of which consists of GO biological process terms annotated to each gene. The Tanimoto score between two genes is simply the ratio of an interaction over a union between two sets. The functional similarity between genes was graphically organized into a network using Cytoscape (http:/www.cytoscape.org/), and functional annotation for each cluster in the network was analyzed using DAVID (https://david.ncifcrf.gov/).

### Validation screen

A validation screen was performed to identify false positives. siRNAs from SMARTpools were rescreened using the same transfection protocol of 10 nM by reverse transfection using Lipofectamine RNAi Max (Thermo Fisher Scientific) according to the manufacturer’s instructions. Moreover, to achieve mitochondrial activity for fluorescence imaging, cells were incubated with 100 nM MitoTracker Red (ThermoFisher, Carlsbad, CA, USA) in growth medium for 30 min. The plates were imaged using a Cytation™ 3 (BioTek Instruments) with a 20x objective.

### Analysis of functional categories

Gene set enrichment analysis (GSEA)^25^ was used to identify significantly enriched functional gene sets using 2D viability per cell measure for A549 and HT29 screening. For the 195 and 109 genes with strong siRNA inhibition in the 2D cell count assay (>2-fold decrease with *p*-value < 0.01), respectively, significantly enriched terms were analyzed using GO biological process terms.

## Supplementary information

Supplementary Figures and Tables

## Data Availability

The screening results of the siRNA library are available at http://compbio.sookmyung.ac.kr/~siTarget.
