# Supplementary material for: Dissecting phenotypic responses of the druggable targetome in cancers
Source: Sci Rep. 2019 Aug 29;9:12513. doi: 10.1038/s41598-019-48989-2 (PMC6715751; doi:10.1038/s41598-019-48989-2)
Supplement: Supplementary file 1 — Supplementary Figures and Tables [file 41598_2019_48989_MOESM1_ESM.pdf]

Supplementary Materials for:

Dissecting phenotypic responses of the druggable targetome in cancers

Euna Jeong, Choa Park, Sung Ung Moon, Juyeon Cho, Mee Song, Seungeun Ryoo, Hyejeong Gu, Yejin Lee, Wooyoung Kim and Sukjoon Yoon

Supplementary Table 1. List of siRNA hits. Log_2 FC means log_2-transformed of readouts.

A549 2D count hits

Gene	log_2 FC	*p*-value	Gene	log_2 FC	*p*-value	Gene	log_2 FC	*p*-value
GPI	-1.367	2.59E-26	ST6GALNAC6	-1.115	1.99E-20	PSMC3	-1.987	1.91E-32
PNPO	-1.089	6.76E-22	ONECUT2	-1.737	1.47E-29	SRD5A3	-1.085	6.54E-21
FOXP2	-1.581	4.16E-28	EFTUD2	-1.279	6.15E-24	XPO1	-1.830	1.96E-31
RAB7A	-1.061	3.20E-21	JAG1	-1.305	3.22E-24	ODC1	-1.365	6.12E-26
TUBB4Q	-2.359	1.52E-35	EXOSC9	-1.760	1.16E-27	B3GNT5	-1.032	2.47E-20
RRM2	-3.171	6.93E-43	ASGR1	-1.713	1.00E-26	GLRX	-1.069	2.07E-21
EIF5	-1.096	5.00E-22	EIF3C	-1.809	2.00E-28	RGS17	-1.216	3.62E-23
EIF4A3	-1.755	3.23E-31	SHOX2	-1.235	3.63E-21	BBOX1	-1.856	2.95E-31
MEN1	-1.010	6.66E-20	DDX47	-1.405	2.69E-23	EIF2S2	-1.186	2.33E-23
ALAS2	-1.593	2.42E-29	SLC2A6	-1.307	2.96E-22	ILF3	-1.441	1.01E-26
PSMC5	-1.643	1.44E-29	RPA1	-1.647	1.30E-26	CTGF	-1.212	1.65E-23
EIF3G	-1.851	3.95E-32	RAN	-1.499	8.18E-25	SAP30BP	-1.089	3.61E-22
KIF13A	-1.148	4.17E-23	PLCD3	-2.331	8.10E-33	COPS2	-1.003	1.35E-20
NDUFA7	-1.182	1.26E-23	MYBL2	-1.125	6.06E-20	POLR2B	-1.400	2.12E-26
UBA52	-2.068	2.33E-34	XAB2	-1.883	4.80E-30	SMARCA4	-1.230	5.22E-24
NXF1	-1.580	4.14E-29	SLC2A8	-1.692	1.60E-25	GNPDA1	-2.319	5.90E-38
STAR	-1.169	2.05E-23	POLR2H	-1.146	1.39E-20	GLT8D1	-1.314	1.42E-26
SLC13A5	-1.057	1.64E-21	OAT	-1.402	1.20E-23	GJB6	-1.063	1.91E-23
MDN1	-1.056	1.86E-21	MPST	-1.008	1.50E-18	BACH2	-1.521	2.99E-30
BDH1	-1.096	1.61E-21	SLC30A8	-1.245	6.05E-22	MADD	-1.255	5.58E-26
TPM1	-1.336	9.77E-26	SLC14A1	-1.346	1.53E-23	RDH10	-1.371	1.38E-27
CDC5L	-1.263	9.86E-25	TOP2A	-1.064	3.20E-19	TCEB3	-1.063	2.13E-21
TNR	-1.014	1.63E-20	PSMD14	-1.112	5.35E-20	KPNB1	-1.402	5.70E-29
ABCC3	-1.024	4.24E-20	DYNLL1	-1.099	3.77E-19	WARS	-1.047	1.20E-23
MYC	-1.070	7.46E-22	APOBEC3A	-1.307	5.47E-22	TOP1	-1.134	5.80E-25
PGAM5	-1.655	1.07E-28	TAF1A	-1.376	4.23E-24	CANT1	-1.180	3.04E-25
ESR1	-1.350	2.91E-25	MYO1A	-1.012	2.61E-18	HOXC6	-1.003	6.66E-22
JMJD6	-1.122	1.00E-22	CHIT1	-1.139	1.32E-20	FUT7	-1.157	3.07E-25
ISL2	-1.855	1.10E-32	GRHPR	-1.576	6.46E-30	DYNC1I2	-1.256	5.68E-26
KIF11	-2.572	9.71E-39	EIF3H	-1.447	3.64E-28	UTRN	-1.335	2.90E-27

PSMC2	-1.192	4.10E-24	LOC136242	-1.234	1.13E-24	DOLPP1	-1.127	1.02E-24
NDUFA6	-2.420	3.04E-37	NPTXR	-1.425	4.84E-28	RNASE6	-1.351	3.33E-26
DPYSL3	-1.348	2.64E-26	EIF3D	-1.073	7.98E-23	ACOX2	-1.180	1.09E-27
ABCD1	-1.099	1.43E-22	KRT8	-1.004	1.04E-20	CASP8AP2	-1.541	5.49E-31
POLR2C	-1.264	6.40E-25	EIF3B	-1.615	1.57E-30	GJA3	-1.731	4.09E-36
ARL4D	-1.577	2.37E-29	POLR2A	-2.081	8.32E-36	PLD3	-1.087	5.44E-27
BRF1	-1.108	3.36E-23	EIF3F	-1.193	2.16E-24	PLCD4	-2.017	3.56E-38
INSM1	-1.757	6.38E-33	NFKBIL2	-1.753	1.07E-31	POLR2F	-1.550	2.65E-28
MAML2	-1.026	1.39E-21	HOXC13	-1.323	1.09E-26	LAMB3	-1.182	3.62E-24
PSMD7	-1.283	8.15E-27	PDE2A	-1.230	6.22E-24	EIF3E	-1.341	7.40E-27
HSPA5	-1.282	1.65E-26	FADS3	-1.136	6.35E-27	NKIRAS1	-1.898	2.07E-33
LZTR1	-1.505	2.36E-29	PLCB3	-1.953	1.49E-37	DHX29	-1.219	7.99E-25
POLR2I	-1.003	3.19E-22	EIF2S3	-1.040	5.58E-25	CCND1	-1.111	2.34E-23
PLAA	-2.100	1.30E-36	POLR2G	-1.376	2.96E-30	HPSE	-1.127	2.45E-23
EIF1AX	-1.125	8.52E-24	GATM	-1.002	1.53E-23	COX15	-1.006	1.28E-21
PSMC6	-1.622	2.05E-31	CD79A	-1.081	1.31E-25	HEYL	-1.483	1.13E-28
GAD1	-1.479	5.01E-26	KIF13B	-1.317	2.07E-29	NKX2-2	-1.168	9.63E-24
TOP1MT	-1.392	2.53E-25	RAD51	-1.383	3.88E-26	FH	-1.033	2.23E-16
B4GALT3	-1.102	1.16E-20	PBX1	-1.041	5.46E-21	SEC14L1	-1.064	2.30E-23
EIF3I	-1.061	1.47E-20	IL10RA	-1.207	1.21E-23	RHOBTB2	-1.059	4.59E-23
UBL5	-1.465	1.53E-27	HOXA1	-1.161	1.75E-25	CYP2A6	-1.859	2.16E-36
BCL2	-1.511	4.29E-28	EIF4A1	-1.433	2.53E-32	RDH5	-1.003	1.54E-23
POLA1	-1.219	5.74E-24	CDY2B	-1.113	2.51E-28	ZKSCAN2	-1.025	9.02E-24
ABAT	-1.187	7.03E-23	GAA	-1.226	3.34E-30	RXRB	-1.192	1.77E-27
NR4A2	-1.029	1.02E-19	FOXD1	-1.338	1.15E-28	RPS2	-2.382	7.95E-40
LBR	-1.109	1.16E-22	GNL2	-1.180	1.50E-29	B4GALT2	-1.142	2.87E-26
ATP6V0D2	-1.004	4.74E-19	PSMC1	-1.696	9.77E-31	PGR	-1.624	2.70E-33
BIRC5	-2.708	1.94E-38	RORC	-1.117	1.75E-28	SLC22A5	-1.396	1.32E-30
SARS	-1.008	1.63E-21	GDF15	-1.371	9.64E-32	CD27	-1.083	6.43E-26
POLE	-1.049	3.85E-22	ALOX15B	-1.576	3.58E-35	PPOX	-1.059	2.32E-25
GFER	-2.275	1.57E-35	SNRNP200	-1.262	4.27E-30	ABCE1	-1.011	1.34E-24
SNW1	-1.386	2.02E-26	WT1	-1.282	9.35E-29	DDX19B	-1.410	6.28E-31
FAAH	-1.420	4.98E-27	PCNA	-1.488	1.54E-30	HOXA7	-1.520	1.43E-31
POMP	-1.192	1.54E-23	AMD1	-1.413	4.15E-33	FCGR2B	-1.688	1.90E-34
DGAT2	-1.143	2.00E-26	RPL4	-2.612	5.03E-42	ERCC1	-1.173	1.48E-25

A549 2D viability hits

Gene	log$_2$ FC	*p*-value	Gene	log$_2$ FC	*p*-value	Gene	log$_2$ FC	*p*-value
RRM2	-2.140	1.04E-34	GAD1	-1.076	7.59E-33	GNPDA1	-1.594	7.12E-36
EIF4A3	-2.167	1.66E-42	EXOSC9	-1.131	1.28E-24	EIF4A1	-1.201	2.96E-27
ALAS2	-1.361	7.44E-30	EIF3C	-2.021	3.19E-34	AMD1	-1.062	6.99E-31
EIF3G	-1.994	4.53E-36	RAN	-1.551	7.27E-32	PLCD4	-1.621	3.99E-35
NDUFA7	-1.016	1.74E-25	PLCD3	-2.100	7.64E-36	POLR2F	-1.141	1.31E-26
UBA52	-2.149	1.28E-34	XAB2	-1.836	3.93E-43	EIF3E	-1.194	2.20E-28
NXF1	-1.593	8.43E-34	SLC2A8	-1.106	4.99E-22	NKIRAS1	-1.234	1.15E-28
PGAM5	-1.564	8.20E-32	EIF3B	-1.474	3.71E-36	DHX29	-1.181	8.65E-26

ESR1	-1.039	5.28E-25	POLR2A	-2.812	1.09E-49	RPS2	-1.414	2.47E-34
ISL2	-1.618	4.41E-27	GFER	-1.440	1.68E-32	DDX19B	-1.535	8.70E-39
KIF11	-1.234	1.14E-21	PSMC3	-1.004	2.36E-27	FCGR2B	-2.114	1.18E-39
NDUFA6	-1.028	4.10E-23	ODC1	-1.036	1.87E-20	RPL4	-2.118	1.29E-36
ARL4D	-1.455	1.52E-31	POLR2B	-1.060	5.98E-24			
PLAA	-1.605	1.42E-34	SMARCA4	-1.007	4.17E-24			

HT29 2D count hits

Gene	$\log_2$ FC	*p*-value	Gene	$\log_2$ FC	*p*-value	Gene	$\log_2$ FC	*p*-value
PNPO	-1.001	6.22E-17	NLGN4X	-1.306	2.46E-22	ARHGEF7	-1.102	2.07E-18
TUBB4Q	-2.368	5.38E-35	MYBL2	-1.179	5.07E-19	NUP153	-1.082	4.09E-18
RRM2	-2.325	1.69E-34	XAB2	-2.056	1.42E-29	NUTF2	-1.515	2.06E-24
NEDD8	-1.234	1.65E-18	POLR2H	-1.519	9.26E-24	KPNB1	-1.335	2.00E-22
EIF4A3	-1.899	2.17E-30	TOP2A	-1.875	4.17E-27	NAGPA	-1.048	2.35E-18
ALAS2	-1.000	2.07E-17	PSMD14	-1.870	8.38E-28	SLC25A37	-1.177	7.41E-20
PSMC5	-2.119	2.73E-32	ITGB5	-2.723	3.95E-35	CAD	-1.289	1.83E-21
DDB1	-1.184	3.02E-21	LOC136242	-1.594	1.79E-25	MYO18B	-1.245	1.01E-20
EIF3G	-3.233	2.56E-41	EIF3B	-2.250	2.34E-32	POLR2D	-1.318	7.22E-22
HSF1	-1.167	9.06E-21	POLR2A	-2.288	1.27E-32	EEF2	-1.258	1.53E-20
UBA52	-2.396	7.29E-35	AGPAT2	-1.001	1.59E-16	CASP8AP2	-1.035	6.06E-18
NXF1	-1.995	5.44E-31	EIF3F	-1.523	1.25E-22	EIF4A1	-1.705	3.01E-28
STAR	-1.516	1.68E-25	VCP	-1.728	4.25E-27	EIF2S1	-2.231	1.35E-33
CDC5L	-1.745	7.56E-29	RPUSD2	-1.242	5.76E-18	BAT1	-1.262	5.81E-23
MYH4	-1.285	2.93E-19	NAT13	-1.372	3.34E-24	TGFB1	-1.362	6.51E-24
PGAM5	-2.000	2.18E-32	PLCB3	-1.619	7.29E-26	PSMC1	-1.990	4.67E-32
BDP1	-1.196	6.70E-22	EIF2S3	-1.528	1.95E-25	ECT2	-1.363	8.29E-25
SETD8	-1.284	5.40E-24	PSMC4	-1.695	1.68E-28	BCAS2	-1.119	1.72E-20
JMJD6	-1.192	3.53E-22	POLR2G	-1.719	9.98E-29	SNRNP200	-1.639	3.24E-28
KIF11	-1.870	1.20E-30	SLC6A7	-1.619	1.43E-27	PHB2	-1.200	3.86E-22
PSMC2	-2.020	9.29E-33	EIF3A	-1.119	1.22E-18	FADD	-1.335	5.07E-24
POLR2C	-1.292	3.08E-24	BTF3	-1.882	1.28E-29	PLCD4	-1.109	1.36E-20
TCP1	-1.221	4.22E-22	UBL5	-1.347	5.68E-26	POLR2F	-1.828	4.07E-29
BRF1	-1.594	2.55E-26	POLA1	-1.241	2.02E-27	LAMB3	-1.058	7.82E-18
CENPE	-1.149	3.13E-15	CSE1L	-1.343	1.80E-32	TXNDC6	-1.319	3.36E-21
CDO1	-1.512	5.69E-24	DPAGT1	-1.288	2.17E-30	EIF3E	-1.429	1.95E-24
PSMD7	-2.289	1.22E-30	BIRC5	-2.531	1.22E-46	ARSK	-1.042	8.70E-19
HSPA5	-2.106	4.60E-31	SNW1	-1.727	1.12E-37	POLR2E	-1.611	2.65E-26
POLR2I	-1.427	1.20E-24	POMP	-1.791	2.32E-38	PADI2	-1.146	1.66E-19
PSMC6	-2.063	3.38E-31	CCT7	-1.064	5.64E-29	NKIRAS1	-1.245	6.63E-21
DHX15	-1.141	3.31E-20	PSMC3	-2.037	6.69E-42	ITGAV	-2.787	3.55E-37
BLM	-1.017	2.10E-18	XPO1	-1.088	2.18E-18	DHX29	-2.442	1.62E-32
EIF3I	-1.486	3.55E-24	SUPT6H	-1.257	6.35E-21	UBB	-1.694	1.62E-27
SMC2	-1.305	1.88E-21	SAP30BP	-1.528	1.74E-24	UNC5B	-1.098	5.52E-19
PLRG1	-1.384	7.52E-23	ANAPC5	-1.095	2.51E-18	RPS2	-2.694	1.28E-38
EFTUD2	-1.601	1.75E-25	COPS2	-1.040	1.01E-17	AHCTF1	-1.074	4.06E-21
EIF3C	-3.041	3.81E-38	POLR2B	-1.656	7.60E-26	DDX19B	-1.428	2.82E-26

CCT3	-1.071	6.07E-19	SMARCA4	-1.220	4.20E-19	FCGR2B	-1.731	3.72E-30
RPA1	-1.674	2.49E-27	MADD	-1.799	7.56E-28	RPL4	-2.379	1.04E-40
RAN	-1.898	2.37E-28	GTPBP1	-1.122	2.87E-19			

HT29 2D viability hits

Gene	log$_2$ FC	*p*-value	Gene	log$_2$ FC	*p*-value	Gene	log$_2$ FC	*p*-value
TUBB4Q	-1.567	4.78E-29	PSMC6	-1.153	5.28E-30	SAP30BP	-1.106	7.57E-35
EIF4A3	-2.589	1.68E-40	EFTUD2	-1.559	5.32E-44	MADD	-1.185	3.88E-34
EIF3G	-1.682	1.92E-31	EIF3C	-1.815	2.88E-45	KPNB1	-1.009	1.07E-33
UBA52	-1.228	3.04E-26	RPA1	-1.150	2.80E-38	SNRNP200	-1.406	9.98E-40
NXF1	-1.172	3.46E-25	RAN	-1.951	2.60E-44	POLR2F	-1.099	8.22E-38
CDC5L	-1.053	2.80E-23	XAB2	-1.304	1.51E-36	DHX29	-1.210	3.22E-36
PGAM5	-1.482	1.65E-44	POLR2A	-2.336	1.19E-50	RPS2	-1.525	1.64E-37
KIF11	-1.355	1.17E-37	SLC6A7	-1.215	1.15E-34	DDX19B	-1.074	5.88E-30
HSPA5	-1.023	7.20E-29	SNW1	-1.318	2.60E-33	RPL4	-1.455	7.00E-33

U87 2D count hits

Gene	log$_2$ FC	*p*-value	Gene	log$_2$ FC	*p*-value	Gene	log$_2$ FC	*p*-value
TUBB4Q	-2.095	8.03E-14	SLC2A8	-1.068	1.58E-13	ILF3	-1.041	1.63E-18
ETF1	-1.540	1.49E-12	POLR2H	-2.692	4.99E-27	SUPT6H	-2.317	4.94E-25
RRM2	-3.185	2.14E-18	SLC14A1	-1.598	6.88E-22	POLR2B	-2.354	5.97E-32
EIF4A3	-2.515	5.41E-16	PSMD14	-1.296	4.88E-18	SMARCA4	-1.303	3.35E-21
PSMC5	-2.569	9.93E-16	TAF1A	-1.010	3.72E-16	GNPDA1	-1.884	9.58E-26
EIF3G	-2.182	1.49E-15	CHIT1	-1.410	4.79E-17	GJB6	-1.214	1.03E-13
UBA52	-2.018	5.30E-13	PHB	-1.171	3.02E-14	MADD	-2.014	3.51E-26
NXF1	-2.375	2.69E-16	DDX24	-1.220	5.00E-17	GTPBP1	-1.046	1.34E-15
STAR	-2.624	3.07E-16	GRHPR	-2.355	5.11E-24	NUP153	-1.538	2.04E-22
CDC5L	-1.324	1.67E-10	LOC136242	-1.732	7.40E-24	POLR1C	-1.161	1.17E-17
PGAM5	-2.886	1.25E-33	EIF3D	-1.089	5.40E-13	KPNB1	-2.211	9.40E-29
IGF2	-1.131	2.51E-19	KRT8	-2.152	3.37E-26	NDUFS5	-1.160	4.42E-17
ARPC4	-1.324	1.95E-20	TGM6	-1.445	3.28E-14	WARS	-1.184	8.75E-15
PLA2G1B	-1.281	1.53E-23	EIF3B	-2.803	1.00E-30	SLC25A37	-1.584	2.92E-21
JMJD6	-2.714	2.50E-31	POLR2A	-3.303	4.20E-37	CAD	-1.157	1.53E-17
ISL2	-1.512	1.06E-25	EIF3F	-1.687	9.60E-25	CANT1	-1.835	5.64E-25
KIF11	-2.956	2.69E-43	HOXC13	-1.296	7.17E-14	MYO18B	-1.538	5.04E-19
PSMC2	-2.279	6.35E-34	VCP	-2.219	1.74E-25	NDUFS1	-1.343	2.59E-18
NDUFA6	-1.890	2.79E-28	HADHA	-1.268	2.72E-06	UTRN	-1.917	4.92E-24
MIOX	-1.841	1.08E-28	PLCB3	-2.751	1.40E-23	RNASE6	-1.161	1.31E-15
NFS1	-1.543	8.32E-28	EIF2S3	-1.212	1.85E-13	HDHD1A	-1.187	7.23E-15
ABCD1	-1.095	6.73E-25	PSMC4	-2.044	3.15E-23	CDY2B	-1.701	3.41E-23
POLR2C	-1.995	1.96E-26	POLR2G	-2.358	3.47E-30	BAT1	-1.078	1.26E-13
ECM1	-1.142	3.93E-17	BCL2L14	-1.345	9.46E-19	PSMC1	-2.957	6.12E-26
PSMD7	-2.103	8.62E-29	POLR2L	-2.169	1.17E-27	SNRNP200	-1.755	2.50E-20
HSPA5	-1.626	4.68E-24	SLC6A7	-1.493	8.01E-23	WT1	-1.019	6.63E-13

LZTR1	-1.703	2.11E-20	RABGGTA	-1.184	2.31E-16	GCNT1	-1.320	1.35E-16
POLR2I	-2.056	4.24E-26	CD79A	-1.291	5.79E-20	PHB2	-1.528	5.00E-18
PLAA	-1.611	1.80E-21	CCT6A	-1.158	1.31E-16	PLCD4	-1.517	5.04E-18
EIF1AX	-1.532	3.44E-19	CSF2	-1.277	3.48E-16	POLR2F	-2.592	3.92E-27
PSMC6	-2.201	2.91E-27	RAD51	-2.449	3.67E-30	ASAH1	-1.983	5.22E-23
NDUFA9	-1.208	1.66E-17	LRAT	-1.379	3.72E-13	MNX1	-1.134	1.19E-13
GAD1	-1.099	6.81E-18	TNFRSF10A	-1.141	1.01E-17	TXNDC6	-1.203	1.20E-14
TOP1MT	-1.206	1.07E-13	BCL2	-1.038	8.04E-15	EIF3E	-1.034	3.21E-14
EFTUD2	-1.532	6.59E-22	ABAT	-2.072	1.07E-23	POLR2E	-1.897	2.97E-22
NQO2	-1.118	3.54E-15	CSE1L	-1.457	5.05E-26	MED14	-1.578	7.19E-20
EIF3C	-2.677	5.61E-24	DPAGT1	-1.252	3.25E-20	CYB5R1	-1.467	9.17E-18
SUPT5H	-3.001	1.18E-25	BIRC5	-2.231	1.46E-34	COX15	-1.351	5.26E-16
SFRP1	-1.128	3.88E-14	GFER	-1.829	1.10E-29	HEYL	-1.086	1.20E-12
RPA1	-1.076	4.83E-13	SNW1	-1.525	5.08E-27	UBB	-1.033	6.93E-12
RAN	-2.147	1.91E-22	FAAH	-1.131	8.85E-20	RXRB	-1.445	2.25E-18
NLGN4X	-1.085	6.79E-12	POMP	-1.093	1.16E-20	RPS2	-1.880	3.65E-23
PROP1	-1.116	3.72E-13	PSMC3	-2.111	1.24E-27	TGS1	-1.008	2.11E-13
PLCD3	-1.934	6.16E-22	XPO1	-1.299	4.34E-19	DIO1	-1.325	6.35E-17
APOC1	-1.327	2.30E-15	ALOX12	-1.583	5.72E-16	DDX19B	-1.329	1.34E-16
SPEN	-1.476	1.79E-18	BBOX1	-1.030	6.92E-18	DGAT2	-1.810	6.67E-26
XAB2	-2.081	6.63E-24	EIF2S2	-1.136	1.28E-19	RPL4	-2.128	2.91E-28

U87 3D sphere count hits

Gene	log_2 FC	*p*-value	Gene	log_2 FC	*p*-value	Gene	log_2 FC	*p*-value
EIF4A3	-2.192	3.22E-18	VARS	-1.016	2.79E-12	POLR2B	-1.354	2.48E-23
PSMC5	-1.804	1.43E-20	DDX24	-1.039	6.19E-15	MADD	-1.734	1.36E-18
EIF3G	-1.451	3.03E-16	SCD	-1.486	4.65E-19	KPNB1	-1.960	1.20E-19
UBA52	-2.192	2.63E-13	EIF3B	-1.222	1.30E-16	HMGCS1	-1.137	4.99E-13
NXF1	-1.420	8.29E-18	POLR2A	-1.374	6.92E-15	FECH	-1.105	1.50E-05
PGAM5	-1.192	4.57E-19	NFKBIL2	-1.106	2.18E-13	CDY2B	-1.369	1.80E-17
ARL2	-1.018	2.91E-17	VCP	-1.114	5.93E-15	PSMC1	-2.302	2.59E-26
JMJD6	-1.280	1.00E-18	SREBF2	-1.297	4.14E-13	SNRNP200	-2.521	4.72E-22
KIF11	-1.509	1.92E-23	PLCB3	-1.335	3.20E-18	PHB2	-1.069	3.52E-15
PSMC2	-1.536	1.05E-20	PSMC4	-1.878	5.67E-21	POLR2F	-1.334	2.74E-16
PSMD7	-1.616	4.56E-21	POLR2G	-1.353	1.81E-19	ASAH1	-1.305	1.53E-14
POLR2I	-1.300	5.20E-17	POLR2L	-1.358	1.93E-15	LSS	-1.610	7.36E-18
PSMC6	-1.885	3.47E-20	SLC6A7	-1.229	1.30E-14	TXNDC6	-1.325	5.07E-14
DDX21	-1.173	4.58E-14	RAD51	-1.072	4.87E-14	POLR2E	-1.226	2.65E-14
EFTUD2	-1.985	4.10E-21	FASN	-1.290	7.30E-19	MED14	-1.202	3.73E-13
EIF3C	-1.477	7.13E-24	SNW1	-1.384	3.76E-18	RPS2	-1.964	3.76E-27
RAN	-1.807	1.09E-28	POMP	-1.073	4.43E-16	RPL4	-3.973	2.79E-28
XAB2	-1.760	5.72E-21	PSMC3	-1.901	1.05E-23	XPO1	-1.484	2.82E-22
POLR2H	-1.760	5.72E-21						

OVCAR8 3D sphere count hits

Gene	log_2 FC	*p*-value	Gene	log_2 FC	*p*-value	Gene	log_2 FC	*p*-value
RRM2	-5.355	8.50E-39	GATA3	-1.143	5.90E-18	PSMC1	-1.264	3.14E-15
EIF4A3	-3.312	7.79E-26	SREBF2	-1.741	5.01E-21	POLR2F	-1.348	7.20E-14
KIF26A	-1.160	1.52E-09	PLCB3	-1.598	4.36E-24	ASAH1	-1.360	6.89E-15
PGAM5	-1.860	1.08E-16	POLR2G	-1.202	2.28E-19	NRG2	-1.464	1.56E-15
PSMD7	-1.271	2.55E-21	UBL5	-1.245	1.06E-12	TXNDC6	-1.234	4.05E-14
PLOD1	-1.080	1.61E-18	SUPT6H	-1.763	1.91E-13	UBB	-2.208	1.99E-21
TAF4	-1.091	8.79E-13	MADD	-2.023	2.71E-24	RPL4	-1.213	4.77E-15
SCD	-2.138	1.34E-27	SHANK3	-1.884	1.57E-21	BNIP2	-1.097	6.08E-13
POLR2A	-2.967	4.75E-36						

Supplementary Table 2. List of selected siRNA hits. Log_2 FC means log_2-transformed of readouts.

2D sample-independent hits

Gene	2D Avg.	2D S.D.	Gene	2D Avg.	2D S.D.	Gene	2D Avg.	2D S.D.
TUBB4Q	-2.274	0.155	PGAM5	-2.180	0.635	PLCB3	-2.107	0.582
RRM2	-2.894	0.492	KIF11	-2.466	0.551	BIRC5	-2.490	0.241
EIF4A3	-2.056	0.403	EIF3C	-2.509	0.633	PSMC3	-2.045	0.062
PSMC5	-2.110	0.463	XAB2	-2.006	0.108	PSMC1	-2.214	0.660
EIF3G	-2.422	0.721	EIF3B	-2.223	0.595	RPS2	-2.319	0.411
UBA52	-2.161	0.205	POLR2A	-2.557	0.654	RPL4	-2.373	0.242

2D sample-dependent hits

Gene	2D Avg.	2D S.D.	Gene	2D Avg.	2D S.D.	Gene	2D Avg.	2D S.D.
NDUFA6	-1.590	1.014	ITGB5	-1.401	1.161	EIF2S1	-1.077	1.012
PLAA	-1.235	1.102	GFER	-1.475	1.025	NKIRAS1	-1.024	1.003
SUPT5H	-1.221	1.542	GNPDA1	-1.510	1.049	ITGAV	-1.221	1.367

3D sample-independent hits

Gene	U87_3D log_2 FC	U87_3D *p*-value	OVCAR8_3D log_2 FC	OVCAR8_3D *p*-value
EIF4A3	-2.192	3.22E-18	-3.312	7.8E-26
PSMC5	-1.804	1.43E-20	-0.904	2.4E-10
KIF26A	-0.745	8.13E-10	-1.160	1.5E-09
NXF1	-1.420	8.29E-18	-0.844	5.4E-10
PGAM5	-1.192	4.57E-19	-1.860	1.1E-16
ARL2	-1.018	2.91E-17	-0.637	1.2E-07
JMJD6	-1.280	1.00E-18	-0.315	2.0E-04
KIF11	-1.509	1.92E-23	-0.724	6.4E-07
PSMC2	-1.536	1.05E-20	-0.992	5.5E-09
PSMD7	-1.616	4.56E-21	-1.271	2.6E-21
POLR2I	-1.300	5.20E-17	-0.807	8.5E-14
TAF4	-0.407	0.000197	-1.091	8.8E-13
EFTUD2	-1.985	4.10E-21	-0.684	1.9E-09
XAB2	-1.760	5.72E-21	-0.985	7.3E-14
VARS	-1.016	2.79E-12	-0.527	6.6E-07
PHB	-0.993	7.64E-11	-0.565	3.0E-07
DDX24	-1.039	6.19E-15	-0.297	1.7E-03
SCD	-1.486	4.65E-19	-2.138	1.3E-27

POLR2A	-1.374	6.92E-15	-2.967	4.8E-36
GATA3	-0.439	5.30E-05	-1.143	5.9E-18
VCP	-1.114	5.93E-15	-0.696	1.1E-08
SREBF2	-1.297	4.14E-13	-1.741	5.0E-21
PLCB3	-1.335	3.20E-18	-1.598	4.4E-24
POLR2G	-1.353	1.81E-19	-1.202	2.3E-19
SLC6A7	-1.229	1.30E-14	-0.408	1.2E-05
RAD51	-1.072	4.87E-14	-0.546	3.4E-05
FASN	-1.290	7.30E-19	-0.811	2.4E-09
SNW1	-1.384	3.76E-18	-0.556	1.4E-06
POMP	-1.073	4.43E-16	-0.472	2.4E-05
PSMC3	-1.901	1.05E-23	-0.745	3.4E-09
POLR2B	-1.354	2.48E-23	-0.534	1.5E-06
MADD	-1.734	1.36E-18	-2.023	2.7E-24
HMGCS1	-1.137	4.99E-13	-0.918	1.2E-11
FECH	-1.105	1.50E-05	-0.521	2.4E-08
CDY2B	-1.369	1.80E-17	-0.581	4.9E-08
PSMC1	-2.302	2.59E-26	-1.264	3.1E-15
PHB2	-1.069	3.52E-15	-0.370	8.2E-04
POLR2F	-1.334	2.74E-16	-1.348	7.2E-14
ASAH1	-1.305	1.53E-14	-1.360	6.9E-15
NRG2	-0.697	1.19E-07	-1.464	1.6E-15
TXNDC6	-1.325	5.07E-14	-1.234	4.1E-14
POLR2E	-1.226	2.65E-14	-0.279	1.5E-02

3D sample-selective hits

Gene	U87_3D $\log_2$ FC	U87_3D *p*-value	OVCAR8_3D $\log_2$ FC	OVCAR8_3D *p*-value
RRM2	-0.981	1.5E-13	-5.355	8.5E-39
EIF3G	-1.451	3.0E-16	0.112	1.4E-01
UBA52	-2.192	2.6E-13	-0.558	1.1E-05
PLOD1	0.157	3.1E-02	-1.080	1.6E-18
PSMC6	-1.885	3.5E-20	-0.402	8.3E-07
DDX21	-1.173	4.6E-14	-0.075	2.4E-01
EIF3C	-1.477	7.1E-24	-0.178	7.1E-02
RAN	-1.807	1.1E-28	0.129	8.4E-02
POLR2H	-1.760	5.7E-21	-0.379	1.2E-04
EIF3B	-1.222	1.3E-16	-0.163	5.2E-02
NFKBIL2	-1.106	2.2E-13	0.126	6.6E-02
PSMC4	-1.878	5.7E-21	0.057	2.4E-01
POLR2L	-1.358	1.9E-15	0.091	1.3E-01

UBL5	-0.205	9.3E-03	-1.245	1.1E-12
XPO1	-1.484	2.8E-22	-0.069	2.7E-01
SUPT6H	-0.269	9.2E-04	-1.763	1.9E-13
KPNB1	-1.960	1.2E-19	-0.146	9.3E-02
SHANK3	0.039	3.1E-01	-1.884	1.6E-21
BNIP2	0.182	2.9E-02	-1.097	6.1E-13
SNRNP200	-2.521	4.7E-22	-0.483	2.5E-05
LSS	-1.610	7.4E-18	-0.251	2.5E-02
MED14	-1.202	3.7E-13	0.042	3.2E-01
UBB	-0.423	2.3E-04	-2.208	2.0E-21
RPS2	-1.964	3.8E-27	-0.292	8.8E-03
RPL4	-3.973	2.8E-28	-1.213	4.8E-15

Supplementary Table 3. Classification of target genes selected from 2D and 3D screening against cancer cells. The knockdown of 77 genes were observed to inhibit the cell proliferation and/or sphere formation.

Hits	Gene symbol	Description	2D cell count			2D cell viability		3D sphere count	
			A549	HT29	U87	A549	HT29	U87	OVCAR8
2D only hits	EIF2S1	eukaryotic translation initiation factor 2 subunit alpha	-0.338	-2.231	-0.661	0.021	-0.522	-0.063	0.059
	ITGAV	integrin subunit alpha V	-0.602	-2.787	-0.273	-0.103	-0.168	-0.014	-0.103
	ITGB5	integrin subunit beta 5	-0.551	-2.723	-0.928	-0.036	-0.223	0.053	0.160
	NDUFA6	NADH:ubiquinone oxidoreductase subunit A6	-2.420	-0.461	-1.890	-1.028	-0.204	-0.639	-0.410
	NKIRAS1	NFKB inhibitor interacting Ras like 1	-1.898	-1.245	0.071	-1.234	-0.432	-0.226	0.139
	PLAA	phospholipase A2 activating protein	-2.100	0.007	-1.611	-1.605	-0.082	-0.781	-0.372
	SUPT5H	SPT5 homolog, DSIF elongation factor subunit	-0.307	-0.355	-3.001	-0.100	-0.136	-0.302	-0.655
3D only hits	ARL2	ADP ribosylation factor like GTPase 2	-0.736	-0.799	-0.070	-0.210	-0.101	-1.018	-0.637
	BNIP2	BCL2 interacting protein 2	-0.500	-0.074	0.126	-0.223	-0.122	0.182	-1.097
	DDX21	DExD-box helicase 21	-0.868	-0.544	-0.529	-0.129	-0.197	-1.173	-0.075
	FASN	fatty acid synthase	-0.581	-0.423	-0.014	-0.071	-0.054	-1.290	-0.811
	FECH	ferrochelatase	0.002	0.119	0.503	0.071	-0.023	-1.105	-0.521
	GATA3	GATA binding protein 3	-0.168	-0.057	-0.219	-0.006	0.002	-0.439	-1.143
	HMGCS1	3-hydroxy-3-methylglutaryl-CoA synthase 1	-0.312	-0.651	0.072	0.017	-0.085	-1.137	-0.918
	KIF26A	kinesin family member 26A	-0.353	-0.074	-0.222	-0.146	0.043	-0.745	-1.160
	LSS	lanosterol synthase	-0.695	-0.585	-0.289	-0.115	-0.054	-1.610	-0.251
	NRG2	neuregulin 2	-0.786	-0.511	-0.590	-0.429	-0.135	-0.697	-1.464
	PLOD1	procollagen-lysine,2-oxoglutarate 5-dioxygenase 1	-0.495	0.278	-0.111	-0.090	0.008	0.157	-1.080
	SCD	stearoyl-CoA desaturase	-0.394	-0.757	-0.366	-0.112	-0.395	-1.486	-2.138
	SHANK3	SH3 and multiple ankyrin repeat domains 3	-0.034	-0.006	0.137	-0.002	-0.035	0.039	-1.884
	SREBF2	sterol regulatory element binding transcription factor 2	-0.629	0.204	-0.875	-0.240	0.033	-1.297	-1.741
	TAF4	TATA-box binding protein associated factor 4	-0.196	-0.006	-0.174	-0.078	-0.042	-0.407	-1.091
	VARS	valyl-tRNA synthetase	-0.763	-0.443	-0.823	-0.194	-0.120	-1.016	-0.527
Selective 2D and 3D hits	GFER	growth factor, augmenter of liver regeneration	-2.275	-0.320	-1.829	-1.440	-0.136	-0.954	-0.212
	GNPDA1	glucosamine-6-phosphate deaminase 1	-2.319	-0.325	-1.884	-1.594	-0.123	-0.927	-0.329
	JMJD6	jumonji domain containing 6, arginine demethylase and lysine hydroxylase	-1.122	-1.192	-2.714	-0.872	-0.469	-1.280	-0.315
	POLR2H	RNA polymerase II subunit H	-1.146	-1.519	-2.692	-0.881	-0.791	-1.760	-0.379
	SUPT6H	SPT6 homolog, histone chaperone	-0.947	-1.257	-2.317	-0.198	-0.422	-0.269	-1.763

Hits	Gene symbol	Description	2D cell count			2D cell viability		3D sphere count	
			A549	HT29	U87	A549	HT29	U87	OVCAR8
2D and 3D common hits	ASAH1	N-acylsphingosine amidohydrolase 1	-0.735	-0.638	-1.983	-0.483	-0.122	-1.305	-1.360
	CDY2B	chromodomain Y-linked 2B	-1.113	-0.165	-1.701	-0.819	-0.078	-1.369	-0.581
	DDX24	DEAD-box helicase 24	-0.517	-0.785	-1.220	-0.275	-0.340	-1.039	-0.297
	EFTUD2	elongation factor Tu GTP binding domain containing 2	-1.279	-1.601	-1.532	-0.673	-1.559	-1.985	-0.684
	EIF3B	eukaryotic translation initiation factor 3 subunit B	-1.615	-2.250	-2.803	-1.474	-0.972	-1.222	-0.163
	EIF3C	eukaryotic translation initiation factor 3 subunit C	-1.809	-3.041	-2.677	-2.021	-1.815	-1.477	-0.178
	EIF3G	eukaryotic translation initiation factor 3 subunit G	-1.851	-3.233	-2.182	-1.994	-1.682	-1.451	0.112
	EIF4A3	eukaryotic translation initiation factor 4A3	-1.755	-1.899	-2.515	-2.167	-2.589	-2.192	-3.312
	KIF11	kinesin family member 11	-2.572	-1.870	-2.956	-1.234	-1.355	-1.509	-0.724
	KPNB1	karyopherin subunit beta 1	-1.402	-1.335	-2.211	-0.776	-1.009	-1.960	-0.146
	MADD	MAP kinase activating death domain	-1.255	-1.799	-2.014	-0.792	-1.185	-1.734	-2.023
	MED14	mediator complex subunit 14	-0.886	-0.589	-1.578	-0.342	-0.228	-1.202	0.042
	NME9	NME/NM23 family member 9	-0.622	-1.319	-1.203	-0.414	-0.524	-1.325	-1.234
	NXF1	nuclear RNA export factor 1	-1.580	-1.995	-2.375	-1.593	-1.172	-1.420	-0.844
	PGAM5	PGAM family member 5, mitochondrial serine/threonine protein phosphatase	-1.655	-2.000	-2.886	-1.564	-1.482	-1.192	-1.860
	PHB	prohibitin	-0.652	-0.924	-1.171	-0.096	-0.176	-0.993	-0.565
	PHB2	prohibitin 2	-0.535	-1.200	-1.528	-0.183	-0.246	-1.069	-0.370
	PLCB3	phospholipase C beta 3	-1.953	-1.619	-2.751	-0.663	-0.617	-1.335	-1.598
	POLR2A	RNA polymerase II subunit A	-2.081	-2.288	-3.303	-2.812	-2.336	-1.374	-2.967
	POLR2B	RNA polymerase II subunit B	-1.400	-1.656	-2.354	-1.060	-0.898	-1.354	-0.534
	POLR2E	RNA polymerase II subunit E	-0.969	-1.611	-1.897	-0.216	-0.734	-1.226	-0.279
	POLR2F	RNA polymerase II subunit F	-1.550	-1.828	-2.592	-1.141	-1.099	-1.334	-1.348
	POLR2G	RNA polymerase II subunit G	-1.376	-1.719	-2.358	-0.768	-0.785	-1.353	-1.202
	POLR2I	RNA polymerase II subunit I	-1.003	-1.427	-2.056	-0.634	-0.873	-1.300	-0.807
	POLR2L	RNA polymerase II subunit L	-0.749	-0.962	-2.169	-0.173	-0.465	-1.358	0.091
	POMP	proteasome maturation protein	-1.192	-1.791	-1.093	-0.559	-0.570	-1.073	-0.472
	PSMC1	proteasome 26S subunit, ATPase 1	-1.696	-1.990	-2.957	-0.962	-0.948	-2.302	-1.264
	PSMC2	proteasome 26S subunit, ATPase 2	-1.192	-2.020	-2.279	-0.461	-0.736	-1.536	-0.992
	PSMC3	proteasome 26S subunit, ATPase 3	-1.987	-2.037	-2.111	-1.004	-0.995	-1.901	-0.745
	PSMC4	proteasome 26S subunit, ATPase 4	-0.518	-1.695	-2.044	0.000	-0.349	-1.878	0.057
	PSMC5	proteasome 26S subunit, ATPase 5	-1.643	-2.119	-2.569	-0.670	-0.899	-1.804	-0.904
	PSMC6	proteasome 26S subunit, ATPase 6	-1.622	-2.063	-2.201	-0.793	-1.153	-1.885	-0.402
	PSMD7	proteasome 26S subunit, non-ATPase 7	-1.283	-2.289	-2.103	-0.611	-0.952	-1.616	-1.271
	RAD51	RAD51 recombinase	-1.383	-0.772	-2.449	-0.173	-0.085	-1.072	-0.546
	RAN	RAN, member RAS oncogene family	-1.499	-1.898	-2.147	-1.551	-1.951	-1.807	0.129
	RPL4	ribosomal protein L4	-2.612	-2.379	-2.128	-2.118	-1.455	-3.973	-1.213
	RPS2	ribosomal protein S2	-2.382	-2.694	-1.880	-1.414	-1.525	-1.964	-0.292
	RRM2	ribonucleotide reductase regulatory subunit M2	-3.171	-2.325	-3.185	-2.140	-0.819	-0.981	-5.355
	SLC6A7	solute carrier family 6 member 7	-0.743	-1.619	-1.493	-0.289	-1.215	-1.229	-0.408
	SNRNP200	small nuclear ribonucleoprotein U5 subunit 200	-1.262	-1.639	-1.755	-0.943	-1.406	-2.521	-0.483
	SNW1	SNW domain containing 1	-1.386	-1.727	-1.525	-0.264	-1.318	-1.384	-0.556
	TONSL	tonsoku like, DNA repair protein	-1.753	-0.368	-0.861	-0.551	-0.130	-1.106	0.126
	TUBB7P	tubulin beta 7 pseudogene	-2.359	-2.368	-2.095	-0.847	-1.567	-0.851	-0.474
	UBA52	ubiquitin A-52 residue ribosomal protein fusion product 1	-2.068	-2.396	-2.018	-2.149	-1.228	-2.192	-0.558
	UBB	ubiquitin B	-0.889	-1.694	-1.033	-0.623	-0.736	-0.423	-2.208
	UBL5	ubiquitin like 5	-1.465	-1.347	-0.607	-0.659	-0.601	-0.205	-1.245
	VCP	valosin containing protein	-0.814	-1.728	-2.219	-0.415	-0.589	-1.114	-0.696
	XAB2	XPA binding protein 2	-1.883	-2.056	-2.081	-1.836	-1.304	-1.760	-0.985
	XPO1	exportin 1	-1.830	-1.088	-1.299	-0.541	-0.404	-1.484	-0.069

Supplementary Figure 1. Comparative analysis of siRNA hits between cell growth and viability assays. (A) 2D cell count vs. 2D cell viability plot of siRNA screening on HT29 cell line. Empty and filled dark circles represent for siNC and siPLK1, respectively. Cell count hits, viability hits, and common hits were represented in red, green, and yellow, respectively. The criteria were $\log_2$ fold-change < -2 and $\log_2$ fold-change < -1 with $p < 0.01$ for cell count hits and viability hits, respectively. (B) The total cell count was plotted against the viability per cell using HT29 screen data.

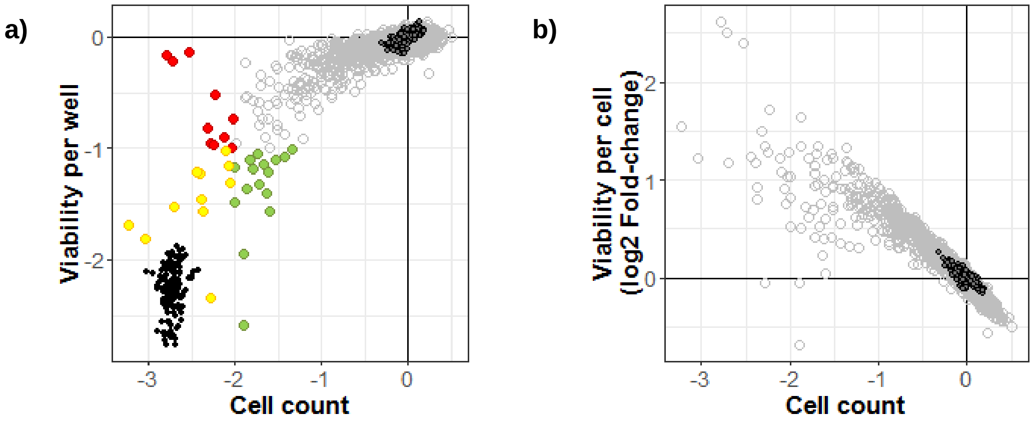

Supplementary Figure 2. Dissecting functional categories of selected siRNA hits from cell count vs. viability measure for HT29. We applied GSEA to the cell count hits to identify significantly cell count enriched and viability enriched gene sets on HT29 cell line. Genes are ranked by the decreased order of the viability per cell measure. Two-sided p-values are used for this plot.

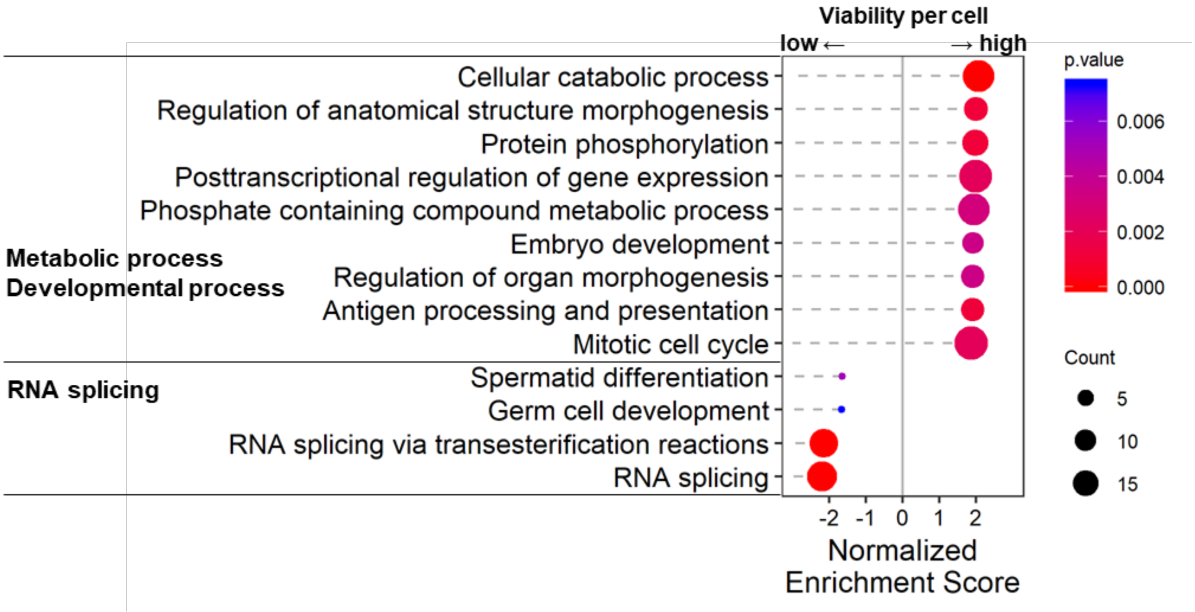

Supplementary Figure 3. Brief summary of data quality of all siRNA screens. Left panel: Screening results are normalized as log2 fold-change values and mean-averaged the three replicates. In the screening results, grey, blue, and red circles represent for siRNA, siNC and siPLK1, respectively. Right panel: Z’-factor was calculated for each plate to quantify the difference of readout between siNC and siPLK1. The range of Z’-factor is from negative infinity to one. Z’-factor of >0.5 generally indicates a high quality assay, while Z’-factor of >0 is considered as a borderline assay (Kumar et al., 2013).

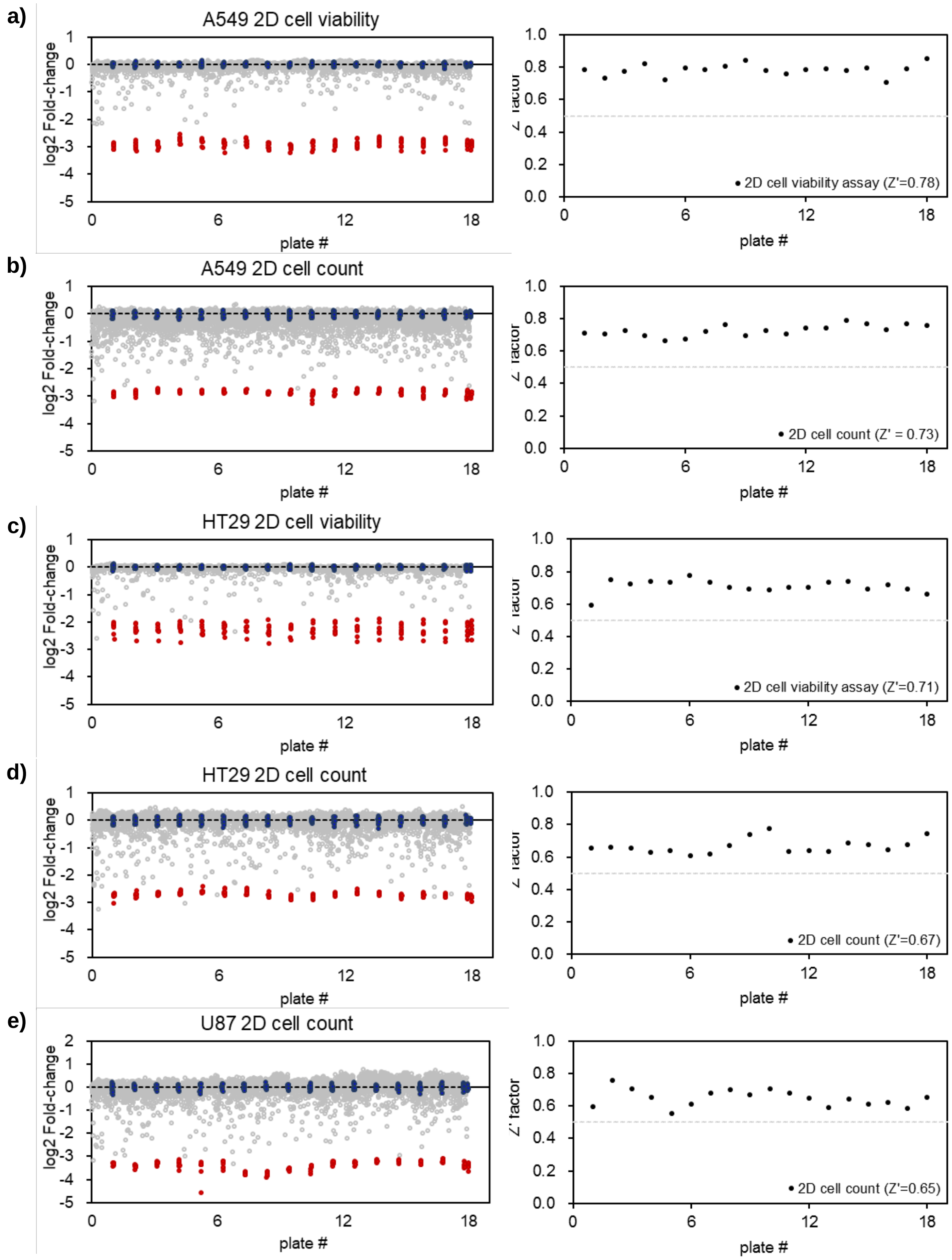

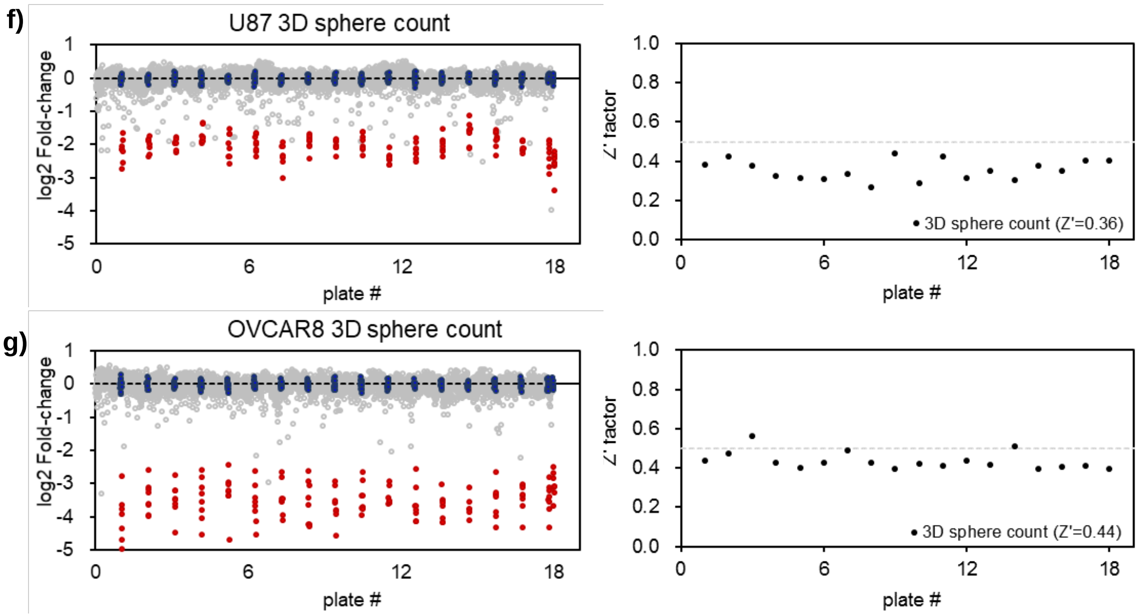

f)
U87 3D sphere count
log2 Fold-change
plate #
Z' factor
3D sphere count (Z'=0.36)
g)
OVCAR8 3D sphere count
log2 Fold-change
plate #
Z' factor
3D sphere count (Z'=0.44)
